# Extirpation of the Larynx

**Published:** 1888-01

**Authors:** 


### EXTIRPATION OF THE LARYNX.

DR. F. DONALDSON, JR., in a letter from Berlin, in *Medical News,* says:

The surgical wards of the Friedrichshain Hospital are under Hahn, who is considered by many to be a greater surgeon than Bergmann. And, indeed, the purpose of this letter is to describe his (Hahn's) last successful operation for extirpation of the larynx. This operation is at present much discussed here in connection with the case of the Crown Prince, about which one hears so much gossip and so little that is reliable. And the German physicians and surgeons, though differing, are at heart one in their sweeping condemnation of Morell Mackenzie. It is currently reported here that from the time he was called to the case, up to a few weeks ago, no German was allowed to examine the Prince's throat; and thatth is was not even permitted to Langraf, who accompanied his highness to England, and remained with him until September 15th.

Much comment has also arisen from Mackenzie calling Krause in consultation, as it is claimed that he does not represent Berlin medicine.

Again, Virchow now declares that he never said that the growth was *not* malignant; only that the *piece* sent him was not. However this may be, the growth is now said to be cancer, and medical Germany rejoices in the triumph of German diagnosis, and declares that the radical operation should have been performed at first, as was advised by Bergmann, Gerhardt, Tobold, etc. But then comes the question, "How far is this operation justifiable? Does it tend to prolong life?" I have before me Hahn's last two papers on the subject:

"Extirpation of the Larynx," and "The Results of Extirpation of the Larynx for Carcinoma," also the record of his 18 operations. The detailed record of those of Bergmann I could not get.

These figures are valuable, for they enable me to give a complete history of this operation till to-day. They are, moreover, entirely reliable, having been kindly given me by the distinguished professor himself.

There have been 103 partial and total laryngectomies done to date, with 21 recoveries. These figures, however, must not be taken as absolute; for in many we have no record beyond that of their supposed recovery at the time the case was reported.

We can obtain a fairer idea of the success of the operation in question by an examination of Hahn's record.

Professor Hahn has classified his operations as follows:

| | No. | No. living. |
|---|---|---|
| I. *Total* extirpation of larynx, on account of carcinoma and sarcoma | 10 | 1 |
| on account of stricture | 2 | 2 |
| II. *Partial* extirpation for cancer | 4 | 3 |
| for stricture | 1 | 1 |
| III. *Partial tracheal* extirpation on account of cancer | 1 | |
| Total | 18 | 7 |

Here, then, we have a total of 7 recoveries in 18 operations. In the partial operations the record is very good; for, as will be seen, we have here a record of 4 recoveries out of 5 operations. I had the pleasure of seeing the only surviving case of *total* extirpation. The patient is now seventy-five years of age, and was operated upon seven years ago. A very beautiful result; the old man having quite a respectable voice, and one can understand every word he says. Professor Hahn does not consider the operation a dangerous one, provided it is performed *early*, and the *cancer* shows no tendency to a *soft* variety.

In the Crown Prince's case, as is known, it was proposed to do a partial extirpation, and, if we can judge anything by the above figures, his chance of surviving was very great. The distress of the German people at his critical condition is very deep, for the Crown Prince is greatly beloved; and I am told that Bergmann had received three letters, from different men, begging that he should take their healthy larynx, if such a thing was possible, and transfer it to the Prince—for they would gladly die to save his life.

Two partial laryngectomies have been done here in the last month: One by Bergmann, upon a healthy man of about forty years, whose larynx had been involved for two months only, and who died a few days after the operation. The second was by Hahn, upon whose invitation I was present at the operation.

The patient was a fine-looking fellow of thirty-six. The cancerous growth, of one and a half years standing, had destroyed the left vocal chord. He was operated upon exactly one month ago. He did well from the first, and is now rapidly recovering. I had the pleasure of seeing him a few days since, sitting up and able to swallow liquid food.

The operation was rapidly performed under perfect antisepsis; the only thing calling for special mention is the use of a large tracheotomy tube,* wrapped with iodoform-gauze. This is of a size which completely fills the lumen of the trachea, and absolutely prevents the entrance of blood into the lungs, besides enabling chloroform to be administered.

The other subject of general discussion here is, or was, the announcement of the discovery of the *bacillus of cancer* by a young man, Scheurlen, an assistant of Leyden.

Scheurlen brought forward the results of his investigation on Monday last at the *Verein für Innere Medicin.* Of course, all medical Berlin was present, and the paper was listened to with great interest, which eventually gave place to greater disappointment. Scheurlen is only twenty-five, and graduated last spring from the University. The tremendous importance of this subject —a cancer bacillus—justifies a *résumé* of the latter's paper, the text of which comes to me this morning in the *Berliner klinische Wochenschrift.* Briefly: He took twenty carcinomas from various sources, and as a vehicle for pure cultures he first used the serum from a pleurisy. This was coagulated in sterilized tubes after Koch's method, and vaccinated with the "cancer milk" from the original cancers—and in three days occurred different changes indicative of the development of bacilli.

From each of ten tumors twenty vaccinations were made, and seven were successful. Microscopic examination of these showed a peculiar bacillus and spores, which, under sufficient power showed movement. The bacilli colored with Gram's method, but only the ends. The spores colored with nitric acid and water only. He claims that no other bacilli and spores have the same form or movement, or color in the same way. The bacilli were found eight times out of ten when colored after Gram's method.

When cultivated from the serum in agar-agar, then bacilli appear in ten hours, the spores after ten days. A first coloring of these spores with fuchsin aniline, and afterward with violet or blue, makes a very striking picture.

In gelatine, the growth of the bacillus is very slow.

Direct cultivation from the tumors in agar-agar or gelatine, gave six results from seventy cultures. Two potatoes gave infusion of peptones. From cabbage, after twelve to twenty-four hours, he got the characteristic microbes.

The mammary glands of six dogs were injected with cultures from potato, or agar-agar. In four days there were circumscribed hardness and swelling of the glands. The tumor was removed from two of the dogs, and microscopic examination showed considerable cell proliferation, with large granular epithelioid cells, among which moving bacilli spores could be seen. Finally, he claimed to have discovered the cause—*i. e.*, the bacillus of cancer.

On this claim the criticisms have been very severe. But it is only fair to await his further investigations.

*An inferior tracheotomy was done.

